# The KT Jeang Prize 2019: Reuben S. Harris: Romancing the Mutator

**DOI:** 10.1186/s12977-019-0486-x

**Published:** 2019-08-29

**Authors:** Retrovirology Editorial

**Affiliations:** London, UK

**Fig. 1:**
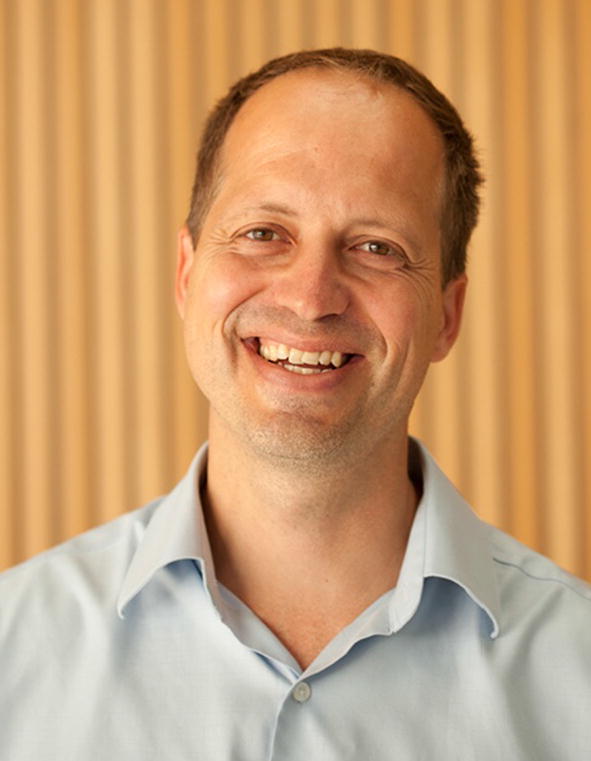
Reuben Harris. Recent photo taken in the Cancer and Cardiovascular Research Building (CCRB) lobby of the at the University of Minnesota

Reuben Harris graduated in 1993 with a BSc in genetics from the University of Alberta (Fig. 1). The degree was distinguished by “specialization” instead of honors because he opted to take a co-ed racquetball and squash course in his senior year. His general interest in sport and competition has been lifelong and joined by a deep interest in mutation research developed during undergraduate studies and, importantly, by taking courses taught by luminaries in the areas of DNA repair, replication, and recombination. In particular, during those years at the University of Alberta, Robert (Jack) von Borstel lectured on mechanisms of mutagenesis and anti-mutagenesis (repair), Linda Reha-Kranz on mechanisms of DNA replication, and Philip (Phil) Hastings and Susan Rosenberg on mechanisms of genetic recombination. These processes combine in intricate ways in real-time and evolutionary-time to generate the vast and amazing diversity of life and adaptability on our ever-changing planet. Captivating topics to say the least for an impressionable young student!

These mentors introduced Harris to many seminal papers on these topics but also, and equally importantly, to model systems and rigorous scientific approaches. During a particularly long discussion one afternoon, von Borstel suggested that Harris (then an undergraduate junior) write to the famous Georg Friedrich (Fritz) Melchers at the Basel Institute for Immunology and inquire about a summer studentship. This idea appealed to Harris and a letter was immediately drafted and sent to Switzerland. A short time later, Melchers responded and offered a position in Antonio Lanzavecchia’s group. Without hesitation, Harris accepted, flew from his hometown of Regina, Saskatchewan to Basel, Switzerland and promptly started the studentship. Lanzavecchia must have sensed a deficiency in immunological training (correctly) and suggested starting with a library submersion to read recent reviews in immunology and learn the basics of this area. Lanzavecchia also provided Harris with his first immunology textbook. After a week or so, this submersion led to a candid discussion over coffee and a challenge to choose between two projects. Harris worked hard over the following months to synthesize peptides and attempt to generate cytolytic T cell responses in cell culture. Although the primary goal was not achieved, Harris learned the importance of positive and negative controls, fumehood ventilation, and open collegiality. The Basel Institute for Immunology was a magical place where students, postdocs, and faculty (called Members) could work closely together to solve important problems. One of the best examples is Susumu Tonegawa’s discovery of the mechanism of VDJ recombination, which is the first essential step in antibody diversification [1, 2].

Upon returning to Edmonton, Canada, Harris started an undergraduate research project with Rosenberg that evolved rapidly into a PhD project. Rosenberg had just been hired as a new assistant professor and was using *E. coli* and phage λ as model systems to test the idea that genetic recombination generates mutations at rates higher than those responsible for the normal background level of “spontaneous” mutation. A few years earlier, John Cairns, Julie Overbaugh, and Stefan Miller published an article titled “The Origin of Mutants” that reignited the Lamarck versus Darwin debate [3]. Lamarckian doctrine suggests that a selective environment can cause the mutations that enable success in that particular situation, whereas Darwinian doctrine posits that mutations happen (by a multitude of mechanisms including those above) and that the environment simply serves to select variants that are best able to cope. Rosenberg and Hastings proposed that recombination may be responsible for the curious mutations reported by Cairns and coworkers that arose at high rates, without detectable cell division, and apparently in response to the selective pressure such as carbon starvation [4]. Harris set out to test this idea and within months was able show that the mechanism of *lac*^−^ to *lac*^+^ mutation in *E. coli* required genetic recombination during conditions of starvation (where lactose was the sole carbon source) but not during conditions of logarithmic growth (where carbon sources were non-limiting) [5]. This discovery led the unveiling of a completely novel molecular mechanism of mutation—mutagenic DNA break repair—and, importantly, to the general idea that mutational mechanisms can be dramatically different under different conditions, and upregulated by stress responses (and still conform to Darwinian principles) (reviewed by [6, 7]). These concepts are important for a number of areas including the evolution of therapy resistance in bacteria, viruses, and tumors.

The combination of summer research at the Basel Institute for Immunology, PhD studies on the mechanism of adaptive mutation in *E. coli*, and continuous exposure to other mechanisms of genetic diversity and evolution led Harris to consider postdoctoral work in molecular immunology and specifically on the mechanism of antibody somatic hypermutation. At the time, it was known that the somatic mutation frequency in antibody gene variable regions was many logs, perhaps even a million-fold, higher than any other gene in the genome. It was also known that these mutations occurred after VDJ recombination and, importantly, that the molecular mechanism was unknown and a subject of considerable interest and speculation. This latter fact intrigued Harris and, after a comprehensive review of the literature and several interviews, a plan was made to do a 2-part postdoc, starting with Nancy Maizels at Yale University and ending with Michael Neuberger at the Medical Research Council Laboratory of Molecular Biology in Cambridge, England.

Harris spent the bulk of 1998 in the Maizels laboratory. Many experiments were done during this period including a yeast 2-hybrid screen and the construction of an *E. coli*-based reporter system for somatic hypermutation. Equally importantly, Harris continued to review the literature on antibody diversification and, together with Maizels, advanced a model for somatic hypermutation based on homologous recombination [8, 9]. In parallel, Harris competed successfully for a Hitchings-Elion Fellowship from the Burroughs Wellcome Fund. This postdoctoral fellowship was one of the first to provide support for postdoctoral studies as well as additional support for a tenure-track position. This 3-year program expanded competitively into a 5-year Career Award format and continued to be unique by allowing studies to be done in different countries with flexible timelines. This fellowship was career-changing and, to this day, Harris continues to be a huge advocate of grant mechanisms that flexibly support postdoctoral to faculty career transitions.

The initial time in the Neuberger laboratory was focused on getting a handle on the molecular mechanism of somatic hypermutation. Harris’ first project aimed to test the hypothesis that the B-cell tropic virus, EBV, produced an inhibitor of somatic hypermutation based on the fact that some EBV-negative Burkitt’s lymphoma cells lines supported ongoing somatic hypermutation and EBV-positive ones did not [10]. Experimental approaches included systematically assessing individual EBV latency genes and programs, as well as using an unbiased subtractive hybridization method. Both approaches yielded interesting results but not the anticipated mechanism. However, during the course of these studies, Tasuku Honjo’s group reported a number of factors expressed differentially between a murine B cell line cultured normally versus induced for immunoglobulin class switch recombination [11]. One of these factors was activation-induced cytidine deaminase (AICDA or AID) which showed homology to the RNA cytosine editing enzyme APOBEC1. This led to the proposal of an RNA editing model for class switching and, shortly thereafter, two important papers demonstrating a requirement for AID in not only this process but also in somatic hypermutation [12, 13].

**Fig. 2:**
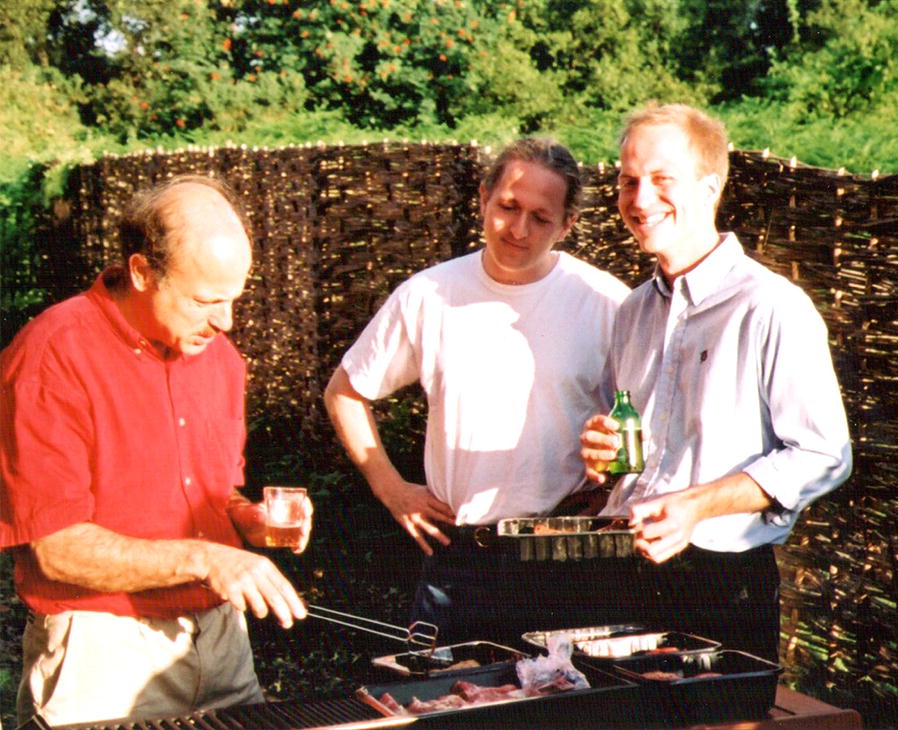
Neuberger lab retreat in Suffolk. Michael Neuberger, Svend Petersen-Mahrt, and Reuben Harris discussing science over BBQ in Suffolk, England

This work stimulated considerable discussion in the Neuberger laboratory including the formulation of a DNA deamination model for antibody diversification by both somatic hypermutation and class switch recombination (reviewed by [14–16]) (Fig. 2). Harris took a degenerate PCR approach to clone human AID from the aforementioned Burkitt’s lymphoma cells lines based on the reported mouse sequence. These efforts quickly yielded the correct cDNA but also a group of related cDNAs shortly thereafter called the APOBEC3s (below). Harris then worked with Svend Petersen-Mahrt to test the first step of the DNA deamination model—namely the idea that AID is a DNA mutator—using *E. coli*-based mutation assays. These experiments clearly showed that AID is a DNA mutator capable of triggering increased mutation frequencies and C to T mutations in purine-C motifs (hotspots) and, importantly, that both of these mutator phenotypes grew synergistically upon ablation of the DNA uracil-specific repair system [17]. Subsequent efforts by other Neuberger lab members and additional groups quickly unraveled many of the steps of the DNA deamination mechanism of antibody diversification. Particularly notable studies were done by Javier Di Noia and Cristina Rada, who used cell lines and murine models to demonstrate that uracil processing by uracil excision repair (UNG2) and mismatch repair (MSH2) were both essential and overlapping secondary steps in both somatic hypermutation and class switch recombination [18–20]. The DNA deamination mechanism of antibody diversification is now an integral part of all immunology text books.

The APOBEC3s were even more intriguing to Harris. Why would human cells have so many APOBEC1-like proteins? Harris and Petersen-Mahrt teamed-up again and showed that several of these enzymes, including APOBEC3G and APOBEC3C, also mutate DNA but, interestingly, caused C-to-T mutations in different dinucleotide contexts (CC and TC, respectively) [21]. In addition, in the same studies, the canonical RNA editing enzyme, APOBEC1, also elicited potent DNA mutating activity. Taken together, these results suggested that the ancestral function of this family of enzymes is likely to be DNA cytosine deamination [22, 23]. A true biological function of these enzymes quickly became apparent with the independent discovery by Michael Malim and coworkers that APOBEC3G (CEM15) inhibits the replication of HIV-1 lacking Vif [24], and subsequent collaborative work by Harris, Kate Bishop, Neuberger, Malim, and coworkers demonstrating a DNA deamination mechanism of retrovirus restriction [25]. Independent studies by other groups near-simultaneously reached the same conclusion [26, 27]. This work was done in 2002 and 2003 as Harris transitioned from a postdoctoral fellowship at the MRC Laboratory of Molecular Biology to an independent faculty position in the Department of Biochemistry, Molecular Biology, and Biophysics Department at the University of Minnesota. Since this time, approximately half of Harris’s papers have focused on the mechanism of HIV-1 restriction (and retrovirus restriction in general) and how this mechanism is counteracted by different viruses. Notable additional discoveries include a role for APOBEC3F in HIV-1 restriction [28], roles for multiple APOBECs in generating the overall G-to-A mutation pattern [28–31], the first structure of APOBEC3G (first for any DNA deaminase with Hiroshi Matsuo [32]), and a surprising essential role for the transcription co-factor CBF-β in Vif-mediated degradation of APOBEC enzymes (collaborative work with Nevan Krogan and John Gross [33]). In addition, over 1500 papers by many groups worldwide have combined since 2003 to elaborate multiple mechanisms of virus restriction by APOBEC3 enzymes and advance a unifying model in which the seven human APOBEC3 enzymes (APOBEC3A-D, F-H) combine to provide an overlapping innate immune defense to exogenous and endogenous viral pathogens (reviewed by [34–36]).

The fact that human cells encode many different DNA cytosine deaminases suggested that one or more of these enzymes may contribute to cancer mutagenesis and tumor evolution [21]. A role for AID in B cell malignancies was inferred quickly based on chromosomal translocations juxtaposing immunoglobulin genes and oncogenes and soon supported by data showing that translocations such as *IgH*-*myc* are AID-dependent in murine models [37, 38]. However, this hypothesis was not trivial to test for the APOBEC3 enzymes and took the better part of a decade including courageous efforts from graduate students in the Harris group. A major advance occurred in 2013 when Michael Burns, Lela Lackey, and coworkers implicated APOBEC3B in breast cancer mutagenesis [39]. APOBEC3B is the only human DNA deaminase family member that is constitutively nuclear [40]. Burns, Lackey and coworkers showed that APOBEC3B is overexpressed in breast cancer cell lines and tumors, is associated with increased mutation frequencies and p53 inactivation, possesses an intrinsic biochemical preference for TC dinucleotides that mirrors cytosine mutation biases in tumors, is capable of inflicting DNA damage responses, and importantly, in breast cancer model systems is required for elevated genomic uracil loads and mutation frequencies [39]. Independent next-generation sequencing studies also implicated an APOBEC family member (not AID) in breast cancer mutagenesis, but it is important to note that these tumor mutation analyses were not accompanied by mechanistic studies to implicate a particular DNA deaminase family member [41]. Subsequent work implicated APOBEC in mutagenesis in many other solid tumor types [42–44]. APOBEC is now appreciated as a major mutation source and therapeutic target in cancer (reviewed by [45, 46]).

The Harris laboratory continues to work on mechanisms of APOBEC-dependent antiviral immunity and cancer mutagenesis with many additional advances including discoveries of the first small molecule inhibitors of APOBEC activity (collaborative work with Daniel Harki [47]), APOBEC-RNA structures (collaborative work with Hideki Aihara [48]), interactions between APOBECs and small DNA tumor viruses polyoma (collaborative work with several groups [49, 50]), and a novel mechanism of APOBEC3B/A counteraction by EBV and related herpesviruses (collaborative work with Lori Frappier and Stephen Rice [51]). Many of these and other advances have been enabled by outstanding local, national, and international collaborations (some not mentioned specifically due to space limitations). Overall interest in APOBEC enzymes in virus restriction and cancer mutagenesis is continuing to grow rapidly with, for instance, breast and lung tumor sequencing studies highlighting prominent roles for APOBEC in driving tumor evolution and promoting metastases [52, 53]. In additional exciting studies, APOBEC is also being harnessed for purposeful genome engineering (base editing) through fusion to CRISPR complexes (reviewed by [54]).

An important part of science are the lessons and legacies passed on from one generation to the next. Harris has no doubt that many of his lab’s present traditions have been directly or indirectly adopted from his mentors over the years. Examples include the keeping of meticulous lab collections of plasmids, oligos, cells, antibodies, etc., champagne for PhD graduation parties, and other beverages for celebratory and sometimes commiserative moments (a particularly vivid example occurred after Harris tore a calf muscle playing soccer and was set on finishing some experiments in the Rosenberg lab before heading to the hospital for additional treatment). Another example is the recruitment of work-hard/play-hard phenotypes who strive to excel at whatever they commit to doing. Harris himself is an international curler and coach, and lab members over the years have exceled at badminton, hockey, brewing, dancing, martial arts, poker, music, and cooking, among many other things. A final example from the Neuberger group is the recruitment of lab members from all parts of the globe to create a novel combination of backgrounds, experiences, and skill sets that will inevitably combine in novel ways to solve current and challenging problems. Diversity is unquestionably vital to the entire scientific process and especially for discovery and innovation.

Harris wishes to acknowledge all of his colleagues and funding over the years and apologizes that specific references could not be made to all. Particularly instrumental support was provided during graduate studies by the Natural Sciences and Engineering Research Council of Canada (NSERC), during postdoctoral studies by the Burroughs-Wellcome Fund (BWF) and Sidney Sussex College, and during faculty years by the US National Institutes of Health (NIAID, NIGMS, and NCI) and the Howard Hughes Medical Institute (HHMI). Notable awards and recognitions include a Governor General of Canada Gold Medal for the best PhD thesis, a non-stipendiary research fellowship at Sidney Sussex College, a NIH MERIT award, and fellowship in the American Academy of Microbiology and the American Association for the Advancement of Science.
